# Editorial: Insights in nuclear medicine: 2022

**DOI:** 10.3389/fmed.2023.1259644

**Published:** 2023-07-26

**Authors:** Giorgio Treglia

**Affiliations:** ^1^Division of Nuclear Medicine, Imaging Institute of Southern Switzerland, Ente Ospedaliero Cantonale, Bellinzona, Switzerland; ^2^Faculty of Biomedical Sciences, Università della Svizzera italiana, Lugano, Switzerland; ^3^Faculty of Biology and Medicine, University of Lausanne, Lausanne, Switzerland

**Keywords:** PET, SPECT, oncology, neurology, cardiology, internal medicine, nuclear medicine, imaging

This Research Topic includes 10 articles related to applications of nuclear medicine techniques in different fields of medicine.

Fluorine-18 fluorodeoxyglucose ([^18^F]FDG) is the most common radiopharmaceutical used for positron emission tomography (PET) and it allows to evaluate the glucose metabolism for oncological and non-oncological indications.

Morland et al. performed a study to construct a PET model to improve lymph node assessment in patients with non-small cell lung carcinoma (NSCLC). A total of 162 NSCLC patients from two centers were included. A model combining visual assessment of lymph node status and tumor maximal standardized uptake values (SUVmax) was selected. The authors demonstrated that primary tumor SUVmax improves lymph node status prediction and could allow a better selection of NSCLC patients who are candidates for minimally invasive approaches (Morland et al.).

Beyond [^18^F]FDG, other PET radiopharmaceuticals evaluating different metabolic pathways can be used for oncological indications as radiolabelled choline which is useful to assess the cell membrane turnover.

The study of Ghidaglia et al. assessed the influence of key histological characteristics on [^18^F]FDG PET and [^18^F]fluorocholine PET positivity in 62 patients with hepatocellular carcinoma (HCC). The authors clearly demonstrated that, among different key histological characteristics, [^18^F]FDG PET and [^18^F]fluorocholine PET positivity appear driven by both the grade and microvascular invasion components in HCC (Ghidaglia et al.).

Another article included in this collection was focused on [^18^F]fluorocholine PET/computed tomography (PET/CT) but for a non-oncological indication. Imperiale et al. investigated the value of presurgical [^18^F]fluorocholine PET/CT in detecting additional hyperfunctioning parathyroids despite a positive [^99m^Tc]Tc-sestamibi parathyroid scintigraphy in 64 patients with primary hyperparathyroidism (pHPT). [^18^F]fluorocholine PET/CT resulted more accurate and useful than [^99m^Tc]Tc-sestamibi scintigraphy in pHPT patients with positive scintigraphic results. Positive parathyroid scintigraphy could be not satisfactory before neck surgery particularly in patients with multiglandular disease. Based on these findings, [^18^F]fluorocholine PET/CT outperforms [^99m^Tc]Tc-sestamibi scintigraphy in detecting hyperfunctioning parathyroid glands and its role in the diagnostic algorithm of pHPT patients should be highlighted.

Another example of non-oncological application of PET imaging is reported by a review article included in this collection which summarizes the potential role and recommendations of [^18^F]FDG PET/CT in bacteremia or bloodstream infections. [^18^F]FDG PET/CT should be considered in suspected complex bloodstream infections, in patients at high risk of metastatic spread, and in bloodstream infection in patients hospitalized in intensive care units. As a matter of fact, in these patients [^18^F]FDG PET/CT has an impact on the management, treatment strategy, and outcome, mainly by directing the diagnostic process ameliorating the detection of infectious foci or by modifying treatment regimens, resulting in reduced relapse and mortality rates. Interestingly, a negative [^18^F]FDG PET/CT has a positive prognostic value and may obviate the need for further workup in patients with bloodstream infections (Hess).

Overall, nuclear medicine imaging techniques are now widely accepted and increasingly used for diagnosis and treatment monitoring of several inflammatory and infectious diseases. An expert opinion article included in this collection written by Glaudemans and Gheysens discuss the current available guidelines on nuclear medicine imaging in infectious and inflammatory diseases, the current limitations of these imaging techniques, and future perspectives of nuclear medicine research for differentiating infection, inflammation and malignancy (Glaudemans and Gheysens).

Among the different PET tracers, radiolabelled fibroblast activation protein inhibitor (FAPI) is emerging both for oncological and non-oncological indications. A case series included in this collection suggested that FAPI PET may be useful for differentiating aseptic loosening from periprosthetic joint infection showing clear advantages over routine examinations (Wang et al.).

Somatostatin receptor PET using radiolabelled somatostatin analogs is currently used for the diagnosis of neuroendocrine tumors (NETs) due to their overexpression of somatostatin receptors. A pilot study from Weissinger et al. explored whether split renal function could be evaluated using imaging data from somatostatin receptor PET/CT performed in 25 patients with NETs prior to peptide receptor radionuclide therapy (PRRT). The authors demonstrated that static somatostatin receptor PET/CT performed at about 30 mins after radiopharmaceutical injection may be used to estimate both split renal function and absolute renal function using the accumulation index (renal parenchyma volume/SUVmean) (Weissinger et al.).

In a preclinical study included in this collection Ding et al. aimed to improve the accuracy of glomerular filtration rate measurement by using Gallium-68 Ethylenediaminetetraacetic acid ([^68^Ga]Ga-EDTA) PET and evaluating its performance in healthy mice and murine models of renal dysfunction. Dynamic [^68^Ga]Ga-EDTA PET provided a reliable and precise means of evaluating renal function in two murine models of renal injury, supporting the possible clinical application of this imaging technique in the near future (Ding et al.).

Beyond PET, nuclear medicine imaging includes also planar scintigraphy and single-photon emission computed tomography (SPECT) techniques which can be used in oncological and non-oncological fields.

The study of Boccalini et al. investigated the effects of manual and semi-automatic methods for assessing semi-quantitative indices in myocardial innervation imaging using iodine-123 metaiodobenzylguanidine ([^123^I]MIBG) scintigraphy in 35 patients with idiopathic Parkinson's disease. The semi-automatic method evaluated by the authors improved the agreement among raters in classifying scintigraphic images as normal or abnormal. These findings have important implications for semi-quantitative assessment of [^123^I]MIBG scintigraphy in clinical routine.

Nuclear medicine does not include only imaging techniques but also therapeutic applications.

Peptide receptor radionuclide therapy (PRRT) is an effective and well-tolerated treatment option for patients with NETs that prolongs progression-free survival. In their retrospective study Trautwein et al. analyzed prognostic risk factors in 62 patients with NETs treated with PRRT. The authors found that PET-based molecular tumor volume at somatostatin receptor PET and chromogranin A in combination were significant prognostic factors for long-term overall survival. Furthermore, an interim somatostatin receptor PET/CT after two cycles of PRRT had the potential in identifying non-responders who may benefit from a change in therapy at an early stage (Trautwein et al.).

In conclusion, the articles included in this Research Topic clearly highlight the bright future of nuclear medicine and radiopharmaceutical sciences.

## Author contributions

GT drafted the manuscript and revised the final version. The author contributed to the article and approved the submitted version.

